# Gender differences in healthy life expectancy among Brazilian elderly

**DOI:** 10.1186/1477-7525-12-88

**Published:** 2014-06-06

**Authors:** Ana Paula Belon, Margareth G Lima, Marilisa BA Barros

**Affiliations:** 1 School of Public Health, University of Alberta, Edmonton, AB, Canada; 2 Department of Public Health, School of Medical Sciences, State University of Campinas, Campinas, São Paulo, Brazil

## Abstract

**Background:**

This study examined gender differences in healthy life expectancy (HLE) and unhealthy life expectancy (UHLE) among people aged 60 years or older living in a large Brazilian city.

**Methods:**

Based on Chiang method, abridged life tables were constructed for men and for women. To calculate HLE, the Sullivan method was applied. Estimates of the prevalence of self-rated health and self-reported functional disability (global, mild/moderate, and severe) were obtained from a population-based household survey carried out in 2008, which involved non-institutionalized individuals.

**Results:**

Findings showed that women live longer and these extra years would be spent in good self-rated health. For example, women aged 60 would live, on average, 4 more years in good health in comparison to men. In terms of global limitations and mild/moderate limitations, no gender differences were detected for HLE. However, UHLE was statistically higher among women than among men at all ages in the global limitations and mild/moderate limitations (except for the age 80). Women at age 60, for instance, could expect to live 3.1 years longer with mild/moderate limitations compared to men. Gender differences were identified for severe limitations for either HLE or UHLE. In comparison to men, women at age 60, for example, would expect to live 2.5 and 2.0 more years without and with severe limitations.

**Conclusions:**

By showing that the advantage of longer life expectancy among women is not necessarily accompanied by worse health conditions, these findings add some evidence to the debate about male-female health-survival paradox. Policy efforts are necessary to reduce gender differences in the quantity and quality of years to be lived, providing equal opportunities to women and men live longer with quality of life, autonomy, and independence.

## Background

Life expectancy in Brazil has increased steadily during the 20th century, rising from 33.7 years in 1900 [1] to 73.5 years in 2010 [2]. However, this considerable improvement has been uneven between men and women. As in several other countries, in Brazil women live longer than men and this gender gap in the life expectancy has widened over time. In 1940, women could expect to live 4.4 years more than men; this difference increased to 7.8 years in 2000 [3]. Although the gender gap had decreased to 7.6 years in 2010 [2], mainly due to the reduction in the homicide rates among men, the mortality differences between sexes are still excessive.

Among older people, the gender differentials in life expectancy have increased over time. Between 2000 and 2010, the gender gap in life expectancy at age 60 widened from 2.9 (18.8 years for men versus 21.7 years for women) to 3.4 years (19.6 years for men versus 23.0 years for women) [2]. Because of this male excess mortality, the accentuated aging process in Brazil has been characterized by an increasing proportion of women aged 60 or older; women constituted 55.5% of the total elderly population in 2010, which was estimated at 20,6 million people [4]. This proportion increases with age, reaching 61.4% of the population aged 80 years or older in 2010 (http://www.sidra.ibge.gov.br/bda/tabela/listabl.asp?c=200&z=t&o=3).

Nevertheless, the female advantage in life expectancy does not necessarily mean that women are healthier than men. Studies using self-report health status measures indicate higher prevalence of functional limitations and poor health among women [5-8], suggesting that the additional years may not necessarily be lived in healthy conditions.

This contrast between female disadvantage in morbidity and female advantage in mortality is known as the male-female health-survival paradox [9-11]. The most common explanations for this paradox are based on biological differences and socio-cultural factors shaping gender roles and, by consequence, health behaviours and use of health services [9,12,13]. Gender differences in self-reported health are another explanation, although there is no evidence for this kind of information bias [14]. Gender differences in terms of survey participation are also considered a factor explaining the differences among men and women; however, a selection bias would have only a small contribution to the male-female health-survival paradox [14]. On the other hand, some studies have questioned this paradox, by showing that the magnitude or even the existence of a female excess morbidity depends on the health indicator and stage of the life course analyzed [15,16].

A way of developing a comprehensive understanding of gender inequalities in mortality and morbidity is to estimate the healthy life expectancy (HLE). By combining information on death and prevalence of healthy and unhealthy states, the total life expectancy is divided into healthy life expectancy (number of years that would be spent in good health) and unhealthy life expectancy (UHLE; number of years that would be lived in poor health). Therefore, this synthetic summary measure adds a qualitative dimension to the life expectancy, identifying in the remaining number of years those that could be lived in healthy or unhealthy conditions.

To date, there are few studies investigating HLE in Brazil [8,17-23]. Most of these studies have pointed out a female advantage in terms of number of healthy years to be lived [21-23] without applying statistical tests to identify if differences between men and women are significant. Therefore, the aim of this study was to estimate gender differences in healthy life expectancy among residents aged 60 years or older in a city in Southeastern Brazil for the year 2008, using self-rated health and self- reported functional disabilities.

We hypothesized that gender differences in UHLE would be significant for both self-reported health and functional disability. Women would expect to live significantly more years in bad health than men due to the combination of their health disadvantage and mortality advantage. In general, women report worse health conditions than men and they are more likely to survive to the older ages when poor self-reported health and functional disabilities are more common [10].

## Methods

### Healthy life expectancy

Healthy life expectancy refers to the number of years that an individual could expect to live in a healthy state, assuming that both mortality and morbidity rates remain constant over time. Since this method combines morbidity and mortality data into a single indicator, it summarizes the length of healthy life.

The concept of HLE has been proposed by Sanders [24] and the first method was developed by Sullivan [25]. The Sullivan method is a prevalence-based method of estimating HLE and UHLE. Another way to estimate HLE is the multistate model, which uses longitudinal data of incidence of health states. Life tables provide the basis to estimate HLE. In the Sullivan method, by applying age-specific prevalence rates of a particular unhealthy state (e.g., poor self-rated health) to a life table function (number of person-years lived in each age interval), the total life expectancy at exact age x is divided into person-years lived with good health (HLE) or poor health (UHLE). Thereby, this method adjusts the total life expectancy for current health status of a population [26].

### Data

A priori, it is worth noting that Campinas is an economically important city in the state of São Paulo and it is the 14th most populous Brazilian city (among 5,570 cities) with a population of 1,080,113 inhabitants in 2010. People aged 60 or older represented 12.4% of the total population in Campinas (http://www.ibge.gov.br).

The data on mortality by sex and age for 2007, 2008, and 2009 were obtained from the Brazilian Mortality Information System (well known as SIM). Since 1989, a project of mortality surveillance in the city of Campinas (developed through a partnership between the Municipal Health Department of Campinas and State University of Campinas) has ensured high quality vital statistics data; only 3% of deaths are assigned to ill-defined causes in this city in 2010. Population estimates for the year 2008 were provided by SEADE Foundation (São Paulo State Data Analysis System Foundation).

Data on health status were derived from the City of Campinas Health Survey (ISACAMP), a health survey carried out in 2008. ISACAMP 2008 was a cross-sectional, population-based household survey, which covered non-institutionalized individuals who were residents in the urban areas of Campinas. The sampling population was comprised of adolescents (10–19 years), adults (20–59 years) and older people (60 years or older). Independent samples of 1,000 individuals were carried out for each of these three age groups. This sample size was calculated considering the estimation of a prevalence of 50%, 95% confidence intervals (95% CI), a sampling error varying from 4% to 5%, and an design effect (deff) of 2.

The sample of households was drawn in two stages. In the first stage, 50 census tracts were selected with probability proportional to the number of households. In the second stage, households within the selected census tracts were sampled. The quantity of households to be visited was defined based on the probability of individuals in each age group living in the household (age structure determined by the demographic census of 2000; http://www.ibge.gov.br). Taking into account a loss of 20% due to closed households and refusals, 2,150, 700 and 3,900 households were selected to interview adolescents, adults, and older people, respectively. The present study analyzed the data from people aged 60 or older (n = 1,519). Every participant signed an informed consent form. Ethical approval was granted by the Research Ethics Committee of State University of Campinas (079/2007).

In the survey, a pre-codified questionnaire encompassing different health topics, (available at http://www.fcm.unicamp.br/fcm/sites/default/files/questionario_ingles.pdf) was applied by trained interviewers. The proxy answers are not frequent among the older people of our sample (5.8%). Four measures of self-reported health status were applied to calculate HLE: self-rated health, global limitations, mild/moderate limitations, and severe limitations. The responses for the self-rated health variable were obtained from this question: “In general, would you say your health is …”. There were five possible answers (“excellent”, “very good”, “good”, “fair”, and “poor”), which were divided into “poor” and “good” self-rated health. “Poor self-evaluation” involved respondents who rated their health as “fair” and “poor”, and “good self-rated” aggregated the other answers.

The questions about functional disability refer to one of the eight scales of SF-36-v2 questionnaire. The SF-36-v1 was translated and validated in Brazil [27] and the second version of this instrument was submitted to validation by Laguardia et al. [28]. We selected four items from the physical functional SF-36 scale in order to evaluate if health conditions limit the following daily activities: walking more than a mile (3 g), walking several hundred yards (3 h), walking one hundred yards (3i), and bathing or dressing (3j). Older people who answered “yes” for the questions 3 g and/or 3 h, but replied “no” for 3i and/or 3j, were considered with mild/moderate limitations. Severe limitations were identified when people answered that their health conditions limit them walking one hundred yards (3i) and/or bathing or dressing themselves (3j). Global limitations refer to people who reported having difficulties to perform any of these activities. The selection of these questions and their operationalization were based on the distribution of the prevalence data in the older population of our sample, as well as on the existent set of Brazilian studies [5,19] that have used similar questions to classify different levels of functional disability.

Prevalence and 95% confidence intervals (95% CI) of poor self-rated health, global limitations, mild/moderate limitations, and severe limitations were calculated by sex and age group (60–64, 65–69, 70–74, 75–79, and 80 years or older). Pearson’s chi-square tests were used to detect significant differences (p-value < 0.05) between age groups and sexes.

### Analysis

To measure the life expectancy for the year 2008, mortality rates were estimated using the average number of deaths for the 2007–2009 triennium and population for 2008. Abridged life tables for 5-year age groups were constructed for men and women based on the Chiang method [29]. From probabilities of death in each age group, the life expectancies at exact ages were calculated. In this study, the estimates were limited to the population at ages above 60 and the final age group was 80 years or older, as mentioned earlier.

Healthy life expectancy was estimated using the method developed by Sullivan [25]. Because the survey has a complex design, weighted prevalence each of the four measuressures of self-reported health status was used [26]. The life expectancy obtained from the abridged life tables for women and men were divided into healthy years and unhealthy years using the age-specific prevalence of self-reported health obtained from ISACAMP. Expected healthy years at each age were calculated by summing years lived in good self-reported health for that age and older ages. The same process was repeated to the other indicators, i.e., prevalence of global limitations, mild/moderate limitations, and severe limitations. Proportion of UHLE represents the relative portion of the life expectancy lived in unhealthy conditions; it was calculated based on the ratio between UHLE and life expectancy at a specific age.

In order to estimate gender inequalities in HLE and UHLE, absolute and relative differences between women and men were calculated. For each indicator, statistical differences between sexes were determined by approximate 95% confidence intervals (95% CI), which were calculated using method described by Jagger et al. [26].

## Results

Among older people, 24.4% (95% CI: 20.0-28.8) rated their health as “excellent” or “very good”, whereas 63.5% (95% CI: 58.7-68.2) reported “good” health. The prevalence of “fair” and “poor” self-reported health were 11.1% (95% CI: 8.9-13.0) and 1.1% (95% CI: 0.6-1.7), respectively. In terms of mobility, 32.4% (95% CI: 25.8-39.7) of older people reported that their health limits them in walking more than one mile or several hundred yards. The self-reported prevalence of difficulties in walking one hundred yards or in bathing or dressing was 10.6% (95% CI: 8.2-13.1) among older people. For global functional disability, the self-reported prevalence was 43.0% (95% CI: 36.5-49.7) (data not shown).

Prevalence rates of poor self-rated health and functional disability by age and sex are presented in Table 1. There was no statistically significant increase in the prevalence of poor self-rated health with age for both sexes. In each age group, differences in this prevalence between men and women were statistically insignificant. The prevalence of global limitations increased with age for men and for women, but significant gender difference was only found in the oldest age group (over age 80). Concerning mild/moderate limitations and severe limitations, the prevalence increased with age for men and for women. While gender differences in the prevalence of mild/moderate limitations were detected at age 75; the differences between men and women in the prevalence of severe limitations were significant in the age groups 70–74 and 80 years and over.

**Table 1:** Prevalence of poor self-rated health and functional disability indicators in the 60-year and older population, by sex and age group (Campinas, 2008)

**Age groups**	**Men**			**Women**			**P-value**^**3**^
	**Total n**	**Prevalence % (95% CI)¹**	**P-value**^**2**^	**Total n**	**Prevalence % (95% CI)¹**	**P-value**^**2**^	
**Poor self-rated health**			0.7503			0.4270	
60	218	9.4 (5.8 - 14.8)		256	10.3 (7.0 - 15.0)		0.7328
65	132	12.9 (7.6 - 20.8)		214	12.3 (7.7 - 18.9)		0.8865
70	117	13.7 (8.5 - 21.4)		164	12.6 (8.5 - 18.3)		0.8102
75	82	14.8 (7.3 - 27.6)		136	16.9 (10.9 - 25.2)		0.7250
80+	66	12.0 (5.6 - 23.6)		134	10.9 (6.4 - 18.1)		0.8248
**Global limitations**			**0.0001**			**0.0000**	
60	218	26.3 (16.6 - 39.0)		256	32.8 (24.9 - 41.9)		0.1984
65	132	28.5 (19.6 - 39.4)		214	37.4 (30.8 - 44.4)		0.0808
70	117	43.0 (32.9 - 53.6)		164	54.2 (43.7 - 64.4)		0.0949
75	82	48.5 (33.9 - 63.5)		136	60.6 (52.2 - 68.4)		0.1107
80+	66	55.3 (41.0 - 68.8)		134	74.1 (64.3 - 82.0)		**0.0060**
**Mild and moderate limitations**			**0.0098**			**0.0000**	
60	218	21.4 (12.2-34.6)		256	26.4 (19.1-35.3)		0.2964
65	132	23.1 (14.9-33.9)		214	31.8 (25.5-38.8)		0.0745
70	117	35.3 (25.1-47.1)		164	37.3 (27.5-48.2)		0.7332
75	82	33.7 (22.0-47.9)		136	48.9 (39.7-58.1)		**0.0499**
80+	66	40.5 (28.0-54.3)		134	43.1 (34.3-52.4)		0.6706
**Severe limitations**			**0.0201**			**0.0000**	
60	218	4.9 (2.4-10.0)		256	6.4 (3.5-11.5)		0.4862
65	132	5.4 (2.4-11.5)		214	5.6 (3.4-9.0)		0.9551
70	117	7.6 (4.1-13.6)		164	17.0 (10.8-25.7)		**0.0258**
75	82	14.8 (7.5-27.0)		136	11.7 (7.1-18.8)		0.5554
80+	66	14.9 (8.4-25.0)		134	31.1 (23.4-39.9)		**0.0089**

^1^95% CI is 95% Confidence Interval.

^2^P-value related to age differences in each sex. Significant differences at the 5% level are indicated in bold type.

^3^P-value related to the differences between sexes in each age group. Significant differences at the 5% level are indicated in bold type.

Table 2 shows gender differentials in life expectancy among older people. Women had longer life expectancy and the gender differences were statistically significant at all ages (confidence intervals were not calculated to the age 80). Despite persistent, the absolute differences in the life expectancy between women and men decreased with age, as expected. The life expectancy at age 60 was estimated in 23.7 years for women and 19.2 years for men, which means a gap of 4.5 years between sexes. The gender difference at age 80 decreased to 2 years.

**Table 2:** Total life expectancy at exact age, by sex (Campinas, 2008)

**Age groups**	**Men**		**Women**		**Gender differences (Women - Men)**
	**LE**	**95% CI**^**1,2**^	**LE**	**95% CI**^**1,2**^	
60	19.2	(18.2 - 20.2)	23.7	(22.8 - 24.6)	4,5*
65	15.6	(14.6 - 16.5)	19.6	(18.8 - 20.5)	4,0*
70	12.4	(11.5 - 13.2)	15.7	(15.0 - 16.5)	3,3*
75	9.4	(8.7 - 10.2)	12.3	(11.7 - 12.9)	2,9*
80+	7.3		9.3		2.0

*Significantly different at the 5% level.

^1^95% CI is 95% Confidence Interval.

^2^Using Chiang’s Method, no confidence intervals were calculated to the age group 80+ .

Healthy life expectancy, unhealthy life expectancy, and proportion of unhealthy life expectancy according to self-rated health and limitations indicators for older men and older women are presented in Table 3. Concerning self-rated health indicator, the HLE at all ages were significantly larger among women than among men. The absolute gender differences in the HLE decreased with age, varying from 4.0 years to 1.9 years. There was no significant difference in the UHLE between men and women.

**Table 3:** Healthy and unhealthy life expectancy at exact age by sex, according to self-rated health and functional disability (Campinas, 2008)

**Age**	**Self-rated health**			**Global limitations**			**Mild and moderate limitations**			**Severe limitations**		
	**Men**	**Women**	**Gender differences (Women - Men)**	**Men**	**Women**	**Gender differences (Women - Men)**	**Men**	**Women**	**Gender differences (Women - Men)**	**Men**	**Women**	**Gender differences (Women - Men)**
**Healthy life expectancy**												
60	16.8	20.8	4.0*	11.8	11.2	-0.6	13.5	14.9	1.4	17.5	20.0	2.5*
65	13.5	17.1	3.6*	8.9	8.3	-0.7	10.5	11.7	1.2	14.0	16.1	2.1*
70	10.7	13.7	3.0*	6.3	5.6	-0.8	7.8	9.0	1.2	10.9	12.3	1.4*
75	8.2	10.7	2.5*	4.5	3.8	-0.7	5.9	6.7	0.8	8.0	9.4	1.3*
80+	6.4	8.3	1.9*	3.2	2.4	-0.8	4.3	5.3	1.0	6.2	6.4	0.2
**Unhealthy life expectancy**												
60	2.4	2.9	0.6	7.4	12.5	5.1*	5.7	8.8	3.1*	1.7	3.7	2.0*
65	2.1	2.5	0.4	6.7	11.4	4.7*	5.1	7.9	2.8*	1.6	3.5	1.9*
70	1.7	2.0	0.4	6.0	10.2	4.1*	4.5	6.7	2.2*	1.5	3.4	1.9*
75	1.2	1.6	0.4	4.9	8.5	3.6*	3.5	5.6	2.0*	1.4	2.9	1.5*
80+	0.9	1.0	0.1	4.0	6.9	2.9*	2.9	4.0	1.1	1.1	2.9	1.8*
**Proportion of unhealthy life expectancy**												
60	12.3	12.3	0.0	38.6	52.7	14.1	29.7	37.3	7.5	8.8	15.4	6.6
65	13.2	12.8	-0.5	42.7	57.9	15.2	32.5	40.1	7.6	10.1	17.8	7.7
70	13.4	13.0	-0.4	48.8	64.6	15.8	36.6	42.8	6.2	12.2	21.8	9.6
75	13.2	13.1	-0.1	52.2	69.1	16.9	37.4	45.2	7.8	14.8	23.9	9.0
80+	12.0	10.9	-1.1	55.3	74.1	18.9	40.5	43.1	2.7	14.9	31.1	16.2

*Significantly different at the 5% level (95% Confidence Interval was estimated only for healthy life expectancy and unhealthy life expectancy).

¹Proportion of unhealthy life expectancy at age x = Unhealthy life expectancy at age x/Life expectancy at age x * 100.

For global limitations, no significant gender differences in HLE were found. In terms of UHLE, women could expect to live more years with limitations than men at all ages. Gender differences in UHLE decreased with age, ranging from 5.1 years at age 60 to 2.9 years at age 80. In comparison to men, women would spend a greater proportion of their remaining lives with limitations; these gender differences increased slightly with age, varying from 14.1% at age 60 to 18.9% at age 80. For both men and women, the proportion of years spent with limitations also increased with age.

With regard to the mild/moderated limitations (Table 3), the differences between sexes were significant for UHLE at all ages, with the exception of age 80. At age 60, women could expect to live 3.1 years longer with mild/moderate limitations than men. At age 75, the gender difference reduced to 2 years. Gender differences in terms of proportion of years spent with mild/moderate limitations decreased with age, varying from 7.5% at age 60 to 2.7% at age 80. Moreover, the proportion of the unhealthy years to be lived with mild/moderate limitations increased with age for men and for women; in both sexes, old-old people aged 80 would spend proportionally more years living with mild/moderate limitations in comparison to young-old people aged 60.

In terms of severe limitations, differences in HLE between sexes were observed at all age groups, except in the oldest age group. Gender differences in HLE decreased with age, varying from 2.5 years at age 60 to 1.3 years at age 75. Unhealthy life expectancy was also higher among women at all ages and the gender gap slightly decreased with age. A woman at age 60 could expect to live 2 years longer with severe limitations than a man at same age. Women would live proportionally more years with severe limitations than men and these gender differences increased with age, ranging from 6.6% at age 60 and 16.2% at age 80. The proportion of years lived with severe limitations nearly doubled between age 60 and age 80 for both sexes. At age 60, men and women would live, on average, 8.8% and 15.4% of their remaining years with severe limitations. From the total number of years to be lived from the age of 80, these proportions increased to 14.9% and 31.1% among men and women, respectively.

## Discussion

Our study uncovered the magnitude and statistical significance of the gender differences in HLE and UHLE in terms of self-rated health and self-report of different levels of functional disability in the 14th largest Brazilian city. To our knowledge, this is the first study in Brazil that analyzed gender differences in HLE and UHLE and estimated the statistical significance of these differences.

Our findings add some evidence to the debate about male-female health-survival paradox [9], by showing that the advantage of a longer life expectancy among women is not necessarily accompanied by worse health. We found that women would live longer and these years would be spent in good self-rated health and without severe limitations; however, they could also expect to live more years with global limitations, mild/moderate limitations, and severe limitations.

It is also worth mentioning that no gender differences in self-rated health were detected in all age groups. For the different levels of functional disability, gender differences were only found among the oldest age groups. These findings support other research that has questioned the female excess in morbidity [9,15,16], by showing the significance of gender differences in health vary according to the health indicator and the stage of the life course. On the other hand, women live longer than men and the gender gap was significant at all ages. This finding is consistent with previous research that has demonstrated the female mortality advantage [9,23]. This relationship between female advantage in life expectancy and a less consistent female disadvantage in morbidity is certainly better elucidated by studies using HLE indicators [11], like ours. Given that previous research has pointed out gender differences in HLE and UHLE varied according to the measure of health status used [9,13], we selected different indicators to explore the male-female health-survival paradox.

In terms of self-reported health, we found that not only women would have a greater length of life, but they would also live more years in good health than men. For instance, at age 60 women could expect to live 4.0 years longer than men in good health conditions. Although these gender differences reduced with age, they are still persistent at the oldest age groups. Differently from previous research [10,21], our study identified similar proportions in terms of years lived in poor self-rated health between men and women. Gender differences in the proportion of UHLE have been attributed to the differences in the self-assessment of health status between men and women [21]. Researchers have suggested that, in addition to biological sex differences, social and cultural factors that shape gender roles may explain the differences in health perception between sexes. Not only female behaviours would be more conducive to good health, but also women would have more knowledge about symptoms and diseases; by consequence, they would be more likely to report poor health conditions [10,13,21]. However, our study did not detect differences in the prevalence of poor self-rated health between older men and older women, and gender differences in HLE were still found with women living more healthy life years than men.

Regarding global limitations and mild/moderate limitations, no gender differences were identified for HLE. Nevertheless, for both indicators UHLE was statistically greater among women than among men at all ages; the only exception was at age 80 when gender differences in UHLE for mild/moderate limitations were not significant. For instance, in comparison to men, women at age 60 could expect to live more than 5.1 years with global limitations; at age 80, the gender difference is smaller (2.9 years) but still significant. Comparisons among studies are difficult due to the selection of different indicators on limitations and variations in wording and scales. Despite these differences, our results are in accordance with other studies, by indicating that women could expect to live a greater proportion of their remaining lives with mild/moderate limitations than men [23,30]. Camargos et al. [19], for instance, verified that proportion of years to be lived with mild (difficult in walking one kilometer) and moderate limitations (difficult in walking 100 meters) was 56.3% and 31.5% among Brazilian women aged 60, respectively. Among men at age 60, the figures were only 38.9% and 21%. Combining mild and moderate limitations indicators (questions about difficulties in walking more than a mile and several hundred yards), our study identified that the proportion of unhealthy years would be 37.3% and 29.7% for women and men aged 60 years, respectively.

With regard to severe limitations, our research pointed out statistically significant gender differences for HLE and UHLE. A 60 year-old woman would expect to live 2.5 years longer without severe limitations than a man at the same age. But, women at age 60 would also live 2 years longer with severe limitations than their men counterparts. That is, despite the advantage of a longer life expectancy, women could expect to live more years with and without severe limitations. In general, mobility indicators and activities of daily living (ADL) indicators, such as ability to dress and bathe, are frequently used to measure severe limitations [5,19]; however, the wide range of conceptualization and operationalization of these indicators hinder comparisons among studies. Despite this diversity of measures and classifications, data from previous studies have suggested that women spend proportionally more years living with severe limitations [19,23,30]. Our data are consistent with these studies, by revealing that the proportion of years lived with severe limitations were higher for women than for men at all ages.

The Sullivan method has some advantages. The first one is its simplicity, since this method only requires cross-sectional data to estimate the HLE. Unlike multistate method, the data availability of morbidity prevalence derived from surveys does not preclude the application of Sullivan method. Another advantage is that Sullivan method can be applied to any state of health, estimating different HLE according to selected measurements of health. As its measure is independent of the size of population and their age structure, HLE is also useful to monitor population health and to make comparative analysis over time or among different populations. Moreover, the index is easily understood, representing the number of healthy and unhealthy years that an individual can expect, on average, to live, as mentioned elsewhere.

Some limitations should be considered when evaluating our results. Firstly, our data came from a survey that did not include people living in institutions; therefore, our results for HLE might be somewhat higher than it would be for the entire population. However, in Brazil, the proportion of older people living in institutions is very small. According to the first Brazilian Census on institutionalized population conducted in 2011 by the National Institute for Applied Economic Research (IPEA) [31], only 0.5% of the total elderly population lives in institutions.

Secondly, due to the small sample size of this survey and lack of information on mortality by socioeconomic indicators, it was not possible to take into account socioeconomic factors that may influence self-reported health conditions in different ways for men and women [16]. Thirdly, although there is some evidence based on the comparisons between subjective and objective measures that women are not more likely to over-report health related problems than men [14], information bias introduced by gender differences in self-reported health may not be ruled out in our study.

Another limitation is that self-reported health status may be affected by cross-cultural differences; therefore, special attention should be paid when comparing our findings with studies conducted in different regions. Nevertheless, it is important to consider that previous studies have shown the validity of self-reported function, by comparing this data with performance measures of physiological impairment [32,33]. In turn, self-rated health is widely recognized as a sensible measure of health conditions that predicts mortality and is associated with clinical measures, engagement in self-care, and healthy lifestyles [34]. The self-rated health is a measure of health status that reflects various dimensions of the everyday life (such as illnesses, functioning, well-being, and life satisfaction [34,35]) and may be affected by engagement in preventive practices, personal skills to cope with diseases and functional disabilities, and performance of social roles in social networks [34,36]. Thereby, aside from providing a broad, full understanding of years lived with quality of life, HLE based on self-rated health is a sensible and irreplaceable indicator because it represents the view of global health status.

On the other hand, our study showed that both older men and older women would spend a smaller proportion of their remaining lives with poor self-rated health, although they would expect to live proportionally more years with limitations. Similar pattern was also found in a Brazilian study that used data from the World Health Survey, which was carried out in 2003 [18]. The low prevalence of self-reported health among older people has been observed in two nationally representative Brazilian surveys. Using Brazilian Household Sampling Survey (PNAD-2003), Dachs & Santos [37] found that only 11.8% and 2.6% of Brazilian people aged 64–85 years had reported poor or very poor health, respectively. Findings from a national telephone survey (VIGITEL - Telephone-Based Surveillance of Risk and Protective Factors for Chronic Diseases) also showed a low prevalence of poor self-rated health (8.0%) among Brazilian people aged 65 years or older [38]. In addition, research has found positive perception of wellbeing [39] and emotional health [6] among older Brazilian people, which may explain the low prevalence of poor self-rated health. In our study, the prevalence of poor self-rated health did not increase with age; this is consistent with findings from a prospective study of a large cohort of Brazilian older people [40]. In contrast to other Brazilian studies that have found that women are more likely to report poor health than men [6,40], our study detected no gender differences. However, the absence of significance may be due to the small sample size of our study.

It is noteworthy that the response rate (for both adults and older people) in our survey was high (85.6%). In the elderly sample, there was a loss of 14.2% (6.1% were refusals, 3.1% were non-contacts and 5.0% were non-response for some other reason). Among older people residing in the selected households, there was a refusal rate of only 5.5% and a loss rate of 6.9% for other reasons; despite the low non-response rates, effects of non-response bias on our results cannot be excluded. However, a recent study conducted in Denmark showed a small contribution of gender differences in terms of selective participation to the explanation of male-female health-survival paradox [14].

A great advantage of our investigation is that it took into account differences in performing some activities of daily living (ADL; ability to “bathe and dress yourself”) and mobility (walking from one hundred yards to more than a mile). In Brazil, most studies have classified functional disability if the respondents reported one or more limitations to perform ADL [20]; others have defined the degree of severity based on the number of answers to questions about ADL [23]. Previous studies have demonstrated the importance of distinguishing mild, moderate, and severe limitations using different questions of ADL and mobility, assuming the functional disability as a continuous and progressive process [5,30].

Considering the paucity of studies analyzing the magnitude of gender differences in HLE [9], our findings may contribute to the debate on male-female health-survival paradox for two reasons. First, by using a single indicator that combines mortality and morbidity, this study revealed the quality of these extra years lived by women. Assessing gender differences in health through an indicator that synthesizes mortality and morbidity rates may shed light into the debate about the consistency of the paradox of women living longer than men but in poorer health conditions [11,15,16]. Second, our study is important because it focused on the magnitude of the gender differences in morbidity, mortality and HLE, instead of analyzing this data for women and men separately [16].

Based on our results, we suggest that more research on gender differences in HLE and UHLE is needed in order to provide a full understanding of the male-female health-survival paradox. Additional step should also be taken towards the application of decomposition techniques to measure the contribution of mortality and morbidity to the gender differences in HLE and UHLE [11,12]. An example of such analysis is the research conducted by Nusselder et al. [11] showing that gender gap in HLE and UHLE in two groups of European countries are masked by gender differences in mortality and morbidity.

Further research investigating the biological, behavioural, and social mechanisms that shape gender differences in life expectancy and HLE may also guide efforts not only to improve male longevity, but also to promote a compression of disability and poor health in the years lived, especially among women.

## Conclusions

In summary, our findings pointed out gender differences in HLE in terms of self-reported health: women would have longer HLE compared to the men, living more years in good health. With regard to global limitations and mild/moderate limitations, our study identified significant differences in UHLE between sexes. Women would also spend more years with and without severe limitations than men. It was also verified that women would live a greater proportion of their lifetime with functional limitations than men. Another important finding in our study is that no gender differences in the prevalence data (except for a few age groups) were detected, while gender differences were found for life expectancy, HLE, and UHLE. This finding is especially important given the debate questioning the male-female health-survival paradox, for which some research has shown no female excess in ill health when using specific health indicators [15,16].

Investigations on gender differences in health among older people are crucial given some facets of population aging, such as premature mortality among men and feminization of old age [41]. Considering that the advantages of a longer life expectancy is only fully achieved if the extra years are lived in good health, investigations on gender differences in HLE are clearly needed in order to provide meaningful insights to the development of action plans that tackle gender inequalities in health and promote healthy, active aging for both men and women. Indeed, more evidence may help inform policies and programs aimed to reduce gender differences in the quantity and quality of years to be lived, providing equal opportunities for women and men to live longer with quality of life, autonomy, and independence.

## Competing interests

The authors declare that they have no competing interests.

## Authors’ contributions

APB conceived the study, estimated the life expectancy and healthy life expectancy, and drafted the manuscript. MGL participated in the conception and drafting of the manuscript, as well as performed the statistical analysis using survey data. MBAB advised on statistical analyses and participated in the revision of the manuscript. All authors read and approved the final manuscript.
